# Quantification and isotherm modelling of competitive phosphate and silicate adsorption onto micro-sized granular ferric hydroxide†

**DOI:** 10.1039/c9ra04865k

**Published:** 2019-07-30

**Authors:** Inga Hilbrandt, Vito Lehmann, Frederik Zietzschmann, Aki Sebastian Ruhl, Martin Jekel

**Affiliations:** Technische Universität Berlin, Chair of Water Quality Control Str. des 17. Juni 135 10623 Berlin Germany Inga.Hilbrandt@tu-berlin.de; TU Delft, Water Management Stevinweg 1 2628 CN Delft Netherlands; Umweltbundesamt Schichauweg 58 12307 Berlin Germany

## Abstract

Adsorption onto ferric hydroxide is a known method to reach very low residual phosphate concentrations. Silicate is omnipresent in surface and industrial waters and reduces the adsorption capacity of ferric hydroxides. The present article focusses on the influences of silicate concentration and contact time on the adsorption of phosphate to a micro-sized iron hydroxide adsorbent (μGFH) and fits adsorption data to multi-component adsorption isotherms. In Berlin drinking water (DOC of approx. 4 mg L^−1^) at pH 7.0, loadings of 24 mg g^−1^ P (with 3 mg L^−1^ initial PO_4_^3−^–P) and 17 mg L^−1^ Si (with 9 mg L^−1^ initial Si) were reached. In deionized water, phosphate shows a high percentage of reversible bonds to μGFH while silicate adsorption is not reversible probably due to polymerization. Depending on the initial silicate concentration, phosphate loadings are reduced by 27, 33 and 47% (for equilibrium concentrations of 1.5 mg L^−1^) for 9, 14 and 22 mg L^−1^ Si respectively. Out of eight tested multi-component adsorption models, the Extended Freundlich Model Isotherm (EFMI) describes the simultaneous adsorption of phosphate and silicate best. Thus, providing the means to predict and control phosphate removal. Longer contact times of the adsorbent with silicate prior to addition of phosphate reduce phosphate adsorption significantly. Compared to 7 days of contact with silicate (*c*_0_ = 10 mg L^−1^) prior to phosphate (*c*_0_ = 3 mg L^−1^) addition, 28 and 56 days reduce the μGFH capacity for phosphate by 21 and 43%, respectively.

## Abstract 2

Adsorption of phosphate onto ferric hydroxide in complex waters is influenced by effects of competition, displacement and surface blockage.

## Graphical abstract

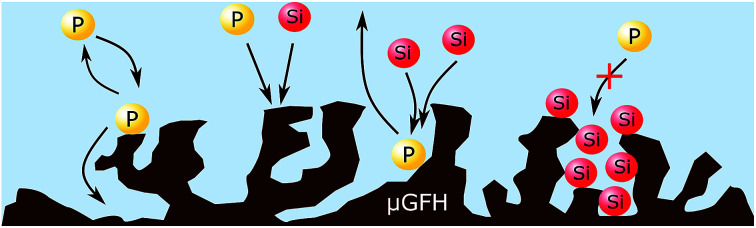

## 1. Introduction

Phosphate loads in up to 20% of European surface waters have to be drastically reduced to reach the good ecological status as demanded by the European water framework directive.^1^ Critical phosphate concentrations for slowly flowing or dammed waters are set as 0.1 mg L^−1^ total phosphorus (TP).^2^ In Germany, the majority of surface waters exceed this value, especially shallow and polymictic lakes. Depending on the lake type, even lower values of 0.02–0.06 mg L^−1^ have been suggested in order to limit algae growth and to reach a good status.^3^

Conventional techniques for P removal include precipitation with di- or trivalent metal ions and biological removal. Low residual phosphate concentrations are achieved with high doses of flocculants. Besides high costs, the advanced treatment leads to an increased input of salts, increased sludge volumes and the need for large sedimentation tanks. A more simple and space saving alternative is the use of adsorption as it is a suitable method to reach low P concentrations.^4^ Many studies on the adsorption of phosphate onto iron oxides have been conducted.^5–8^ Adsorption of phosphate as a polyprotic anion takes place over a wide pH range and has its maximum at values below the pH_PZC_ of the adsorbent.

Adsorbents are not specific for one target substance but accumulate a mixture of competing water constituents. Competitive adsorption of phosphate onto iron hydroxides was widely studied.^6,8–11^ While sulfate and chloride were shown to have no effects^12^ and bivalent cations (*e.g.* Ca^2+^, Mg^2+^) can have beneficial surface complexation effects on phosphate, bicarbonate and silicate proved to have adverse impacts.^13^

Silicate is released during wreathing of Si-containing minerals and occurs in natural waters in concentrations of 3 to 30 mg L^−1^, mainly as Si(OH)_4_.^14^ The sorption of silicate onto iron hydroxides receives ongoing attention.^9,13–19^ The adsorption is pH-dependent with increasing adsorption until the silicate species changes from H_4_SiO^0^_4_ to H_3_SiO_4_^−^ at p*K*_a_ 9.82.^13^ The adsorption is accompanied by a net release of protons, which results in a decrease of the iso-electrical point of the adsorbent surface. At the pH_PZC_ of the adsorbent the building of two different surface complexes is proposed by Hiemstra.^13^ A binuclear bidentate complex with exchange of two ligands resulting in ≡(FeO)_2_Si(OH)_2_ and a mononuclear monodentate complex ≡(FeOHFeO)Si(OH)_3_. At low concentrations and low pH values silicate is mostly present as monomers, whereas the amount of oligomeric and polymeric silicates increases steeply at circum-neutral pH values.^13^ Polymerization was observed at molar ratios of Si/Fe below 0.1.^14,15,20^

In competitive adsorption experiments phosphate was less affected by silicate than oppositely.^13^ While at low pH-values the effect of phosphate on silicate was very large, it was less for higher pH-values. For the competitive adsorption of silicate and arsenate no influence was found for pH values below 9, but silicate was able to replace arsenate when adsorbing onto ferrihydrite above pH 9.^20^ However, polymerization of silicate was prevented when arsenate was added first.^14^ They conclude that polymerization is a surface controlled process. Christl *et al.*^18^ suggest that a long pre-equilibration of the adsorbent with silicate might alter the surface configuration of the adsorbent, favouring the formation of oligomers and polymers and thus reducing reactivity. No binary adsorption isotherms to fit this data have been developed yet to our knowledge.

Given the high relevance of phosphate for surface water quality, the adverse impacts of the ubiquitously present adsorptive competitor silicate requires further elucidation. The current study highlights the effects of variable silicate concentrations, preloading times, and iron-complexation on phosphate adsorption onto a micro-sized iron hydroxide (μGFH, characterized in a previous study^21^). In addition, various bi-solute models are tested for describing the observed competitive adsorption. Further, we also tested the potential reversibility of silicate and phosphate adsorption under practically relevant conditions. The provided results improve the understanding of competitive phosphate–silicate adsorption on iron hydroxide surfaces, thus providing better means for process control and safety.

## 2. Material and methods

### 2.1. Adsorbent

Micro-sized ferric hydroxide was obtained from GEH Wasserchemie (Osnabrück, Germany). Granular ferric hydroxide (GFH) is produced by precipitation of Fe(OH)_3_ from a FeCl_3_ solution and subsequent conditioning of the resulting sludge to obtain stable granules of up to 3 mm grain size.^22^ In the current study, we used only particles <0.3 mm, potentially increasing adsorption kinetics and overall loadings.^23^ Conventional GFH consists to 50–70% of akaganeite (β-FeOOH), ferrihydrite, and other iron oxides.^24^ Saha *et al.*^25^ reported a point of zero charge (pH_PZC_) of 7.5 for GFH. The supplied μGFH material had a water content of approx. 53% and a specific surface area of approx. 300 m^2^ g^−1^ dry weight. Sieve analyses showed a contribution of 30, 25, and 45% weight of the fractions < 63 μm, 63–120 μm and 120–300 μm, respectively, and particle counting with a particle analyser (PAMAS SVSS) revealed that 99% of the particles were smaller than 63 μm.^21^ The μGFH was wet-sieved to the desired grain fractions and then air-dried at room temperature. The residual water content varied between 10 and 20%. Previous experiments showed no alteration of the material due to drying. A detailed characterization of the adsorbent was provided by Hilbrandt *et al.*^21^

### 2.2. Experimental set-up

Batch experiments were conducted at room temperature (20 ± 2 °C) to obtain adsorption isotherms. All chemicals and reagents used were of analytical grade or higher. NaCl was added to deionized water (DI) to set an ionic strength of 10 mmol L^−1^. Initial phosphate and silicate concentrations (added as KH_2_PO_4_ and Na_2_SiO_3_·5H_2_O) were adjusted to 4 mg L^−1^ P and 10 mg L^−1^ Si respectively (unless indicated otherwise). 2 mmol L^−1^ MES (2-(*N*-morpholino)ethanesulfonic acid, p*K*_a_ = 6.1), BES (*N*,*N*-bis(2-hydroxyethyl)-2-aminoethanesulfonic acid, p*K*_a_ = 7.1) or TAPS (*N*-tris(hydroxymethyl)methyl-3-aminopropanesulfonic acid, p*K*_a_ = 8.4) were used to buffer the pH value, adjusted with NaOH. All buffer substances were purchased from Sigma Aldrich (Germany). No significant adsorption of the buffer substances onto μGFH was found in preliminary tests and thus no competition with the target substances was expected. The isotherms were obtained by varying the adsorbent doses between 40 and 500 mg L^−1^. Dry μGFH was added to 100 mL or 150 mL batches of test solutions containing the target substances and shaken at room temperature at 220 rpm. After 72 h, the adsorbent was separated using membrane filtration (0.45 μm, cellulose nitrate), as kinetic experiments showed no further adsorption after that time period.^26^

For desorption experiments the suspensions were centrifuged after 72 h contact time (as described above, without adsorbent removal by filtration), following a method introduced by Aschermann *et al.*^27^ 95 mL of the solution were removed after centrifugation and the same volume of desorption solution was added. The desorption solution corresponded to the adsorption solution without the target substances. The desorption batches were shaken for another 72 h prior to adsorbent removal by filtration and subsequent analyses.

For the influence of silicate in simultaneous adsorption, μGFH was added to the above described test solution containing 3 mg L^−1^ PO_4_–P and varying concentrations of silicate (6, 10 and 18 mg L^−1^). In addition to competitive phosphate–silicate tests in DI-water, drinking water tests were conducted. The concentrations of 6, 10, 18 mg L^−1^ Si were added to drinking water which itself had a Si concentration of 6 mg L^−1^, resulting in final Si concentrations of 12, 16, 24 mg L^−1^. Sequential adsorption experiments were carried out as described for the single solute tests but with the addition of the second substance from a highly concentrated stock solution after a contact time of 72 h. The equilibrium concentrations of both substances were measured after the cumulative adsorption time of 144 h. Further experiments were carried out with 7, 28 and 56 d contact time between silicate and μGFH with subsequent addition of phosphate and additional 7 d of contact time in the binary solution.

### 2.3. Analytical methods

μGFH doses in the batch experiments were gravimetrically controlled by membrane filtration through pre-washed 0.45 μm cellulose nitrate filters, which were dried for 24 h at 105 °C and weighed before and after filtration. Orthophosphate was quantified *via* flow injection analysis according to ISO.^28^ Silicate concentrations were determined photometrically (Lambda 12, PerkinElmer) with test kits (Spectroquant, Merck) using 10 mm glass cuvettes. The uncertainty of the measurement is shown with a 95% confidence interval expressing the summed up errors of the experimental and analytical processes.

### 2.4. Adsorption modelling

#### 2.4.1. Single component isotherms

The adsorption equilibrium in a single component system can be described by isotherm equations of Freundlich (eqn (1)) with the equilibrium loading *q*_*i*_ (mg g^−1^), the Freundlich coefficient *K*_F,*i*_ (L^*n*^ (g mg^*n*−1^)^−1^), the equilibrium concentration *c*_*i*_ (mg L^−1^) and the Freundlich exponent *n*_*i*_ (−), of Langmuir (eqn (2)) with the maximum loading *q*_m,*i*_ (mg g^−1^) and the Langmuir coefficient *K*_L,*i*_ (L mg^−1^) or of Redlich–Peterson (R–P, eqn (3)) with the coefficients *A*_*i*_ (L g^−1^), *b*_*i*_ (L mg^−1^)^*β*^ and *β* (−).

1 *q*_*i*_ = *K*_F,*i*_*c*_*i*_^*n*_*i*_^

2
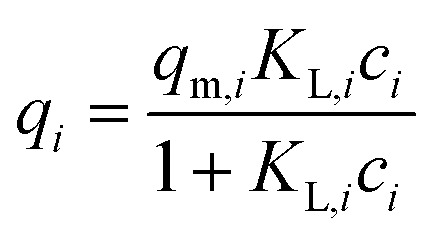
3
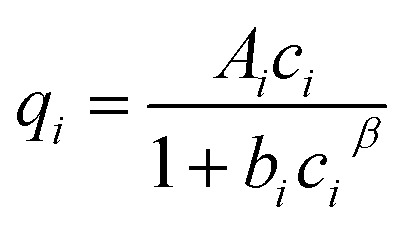


#### 2.4.2. Multi-component isotherms

For the description of adsorption in multi-solute systems, multi-component isotherm equations were developed by extending the above described single component isotherms.^29^ The following equations were used in this work:

(a) Non-modified Langmuir multi-component isotherm (NLMI) with the Langmuir parameters *q*_m,*i*_ and *K*_L,*i*_ derived from the single-component isotherms^30^4
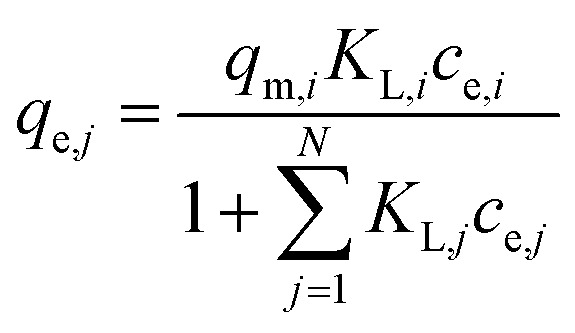


(b) Modified Langmuir multi-component isotherm (MLMI) with the Langmuir parameters *q*_m,*i*_ and *K*_L,*i*_ derived from the single-component isotherms and an additional interaction term *η*_*i*_,^31^ number of fitting parameters: 2 (*n*_*i*_, *n*_*j*_)5
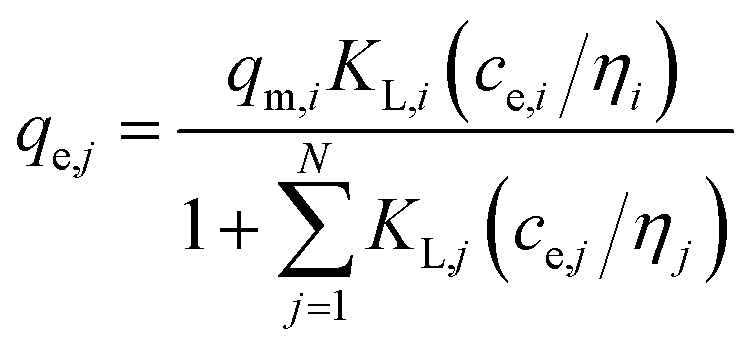


(c) Extended Langmuir multi-component isotherm (ELMI) with a total maximum loading of both components *q*_max_ (mg g^−1^) and Langmuir coefficients *K*_*i*_ and *K*_*j*_ in the binary solution,^32^ number of fitting parameters: 3 (*q*_max_, *K*_*i*_, *K*_*j*_)6
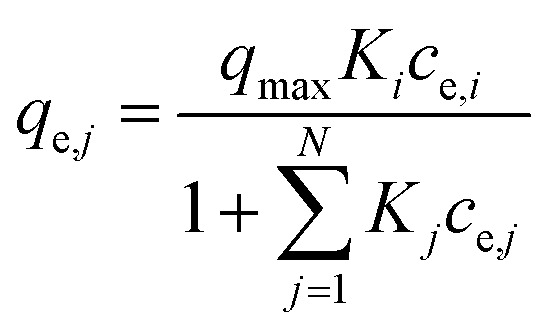


(d) Extended Freundlich multi-component isotherm (EFMI) with the Freundlich parameters *K*_F_ and *n* from the single-component isotherm and the binary coefficients *x*_*i*_, *y*_*i*_ and *z*_*i*_,^31,33^ number of fitting parameters: 6 (*x*_1_, *y*_1_, *z*_1_, *x*_2_, *y*_2_, *z*_2_)7
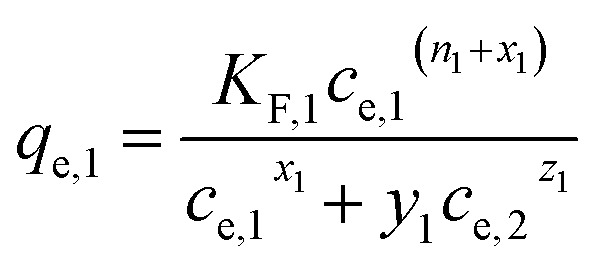
8
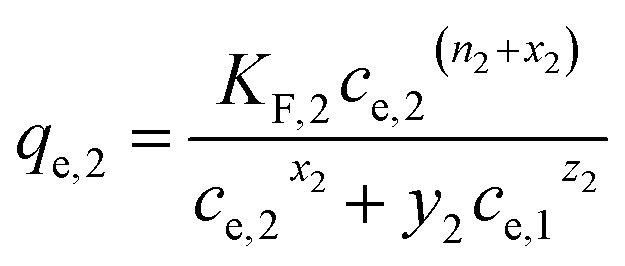


(e) Sheindorf–Rebuhn–Sheintuch equation (SRS) with the Freundlich coefficient from the single-solute isotherm *K*_F_ and the interaction factor *a*_*ij*_,^34^ number of fitting parameters: 2 (*a*_*ij*_, *a*_*ji*_)9
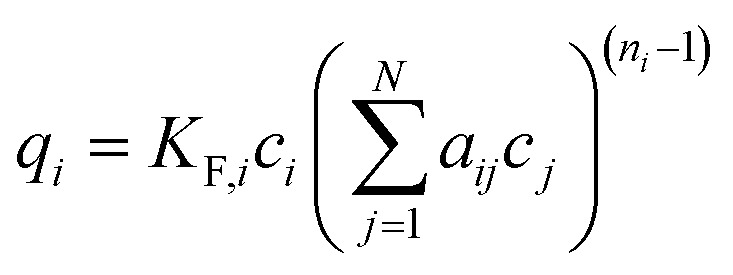


(f) Non-modified Redlich–Peterson multi-component isotherm (NRPMI) with the R–P single-component isotherm parameters *A*_*i*_, *b*_*i*_ and *β*_*i*_^35^10
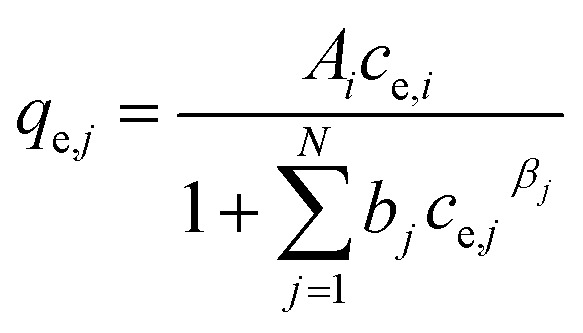


(g) Modified Redlich–Peterson multi-component isotherm (MRPMI) with the single-component isotherm parameters and an additional interaction factor *η*_*i*_,^35^ number of fitting parameters: 2 (*n*_*i*_, *n*_*j*_)11
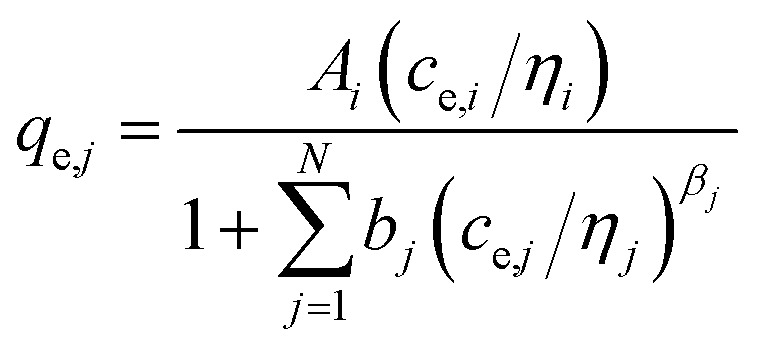


(h) Ideal adsorbed solution theory (IAST) with the mole fraction *z*_*i*_, the spreading pressure *φ*, and the Freundlich parameters *K*_F,*i*_ and *n*_*i*_ and the total loading *q*_T_,^29^ number of fitting parameters: 2 (*φ*, *q*_T_)12
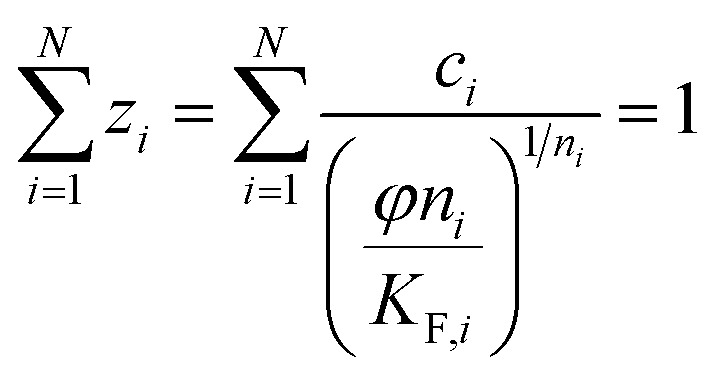
13
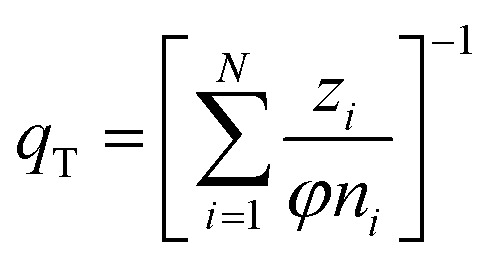


#### 2.4.3. Parameter fitting and determination

All models described above use isotherm parameters derived from single-solute fittings. The parameter fitting was thus applied to the additional parameters for binary systems. The parameters were fitted by minimizing Marquardt's percent standard deviation (MPSD).^36^ The equation incorporates the degree of freedom into the geometric mean error distribution and was used by Srivastava *et al.*^31^ for fitting of binary adsorption isotherms of cadmium and nickel onto bagasse fly ash. Through the incorporation of the degree of freedom a better comparison between models with different numbers of fitting parameters can be assured. *n*_m_ is the number of experimental data points and *n*_p_ the number of parameters in the isotherm equation.14
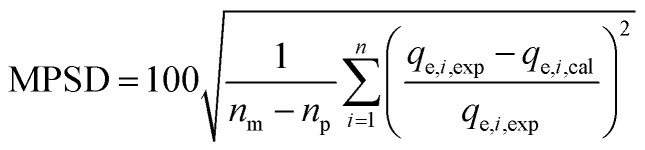


To avoid finding results related to a local minimum of the minimization criteria a matrix of starting values containing at least 500 different combinations of the parameters was used. Thus, it was made sure to find the global minimum and the best fitting parameters.

## 3. Results and discussion

### 3.1. Effect of pH

**Fig. 1:**
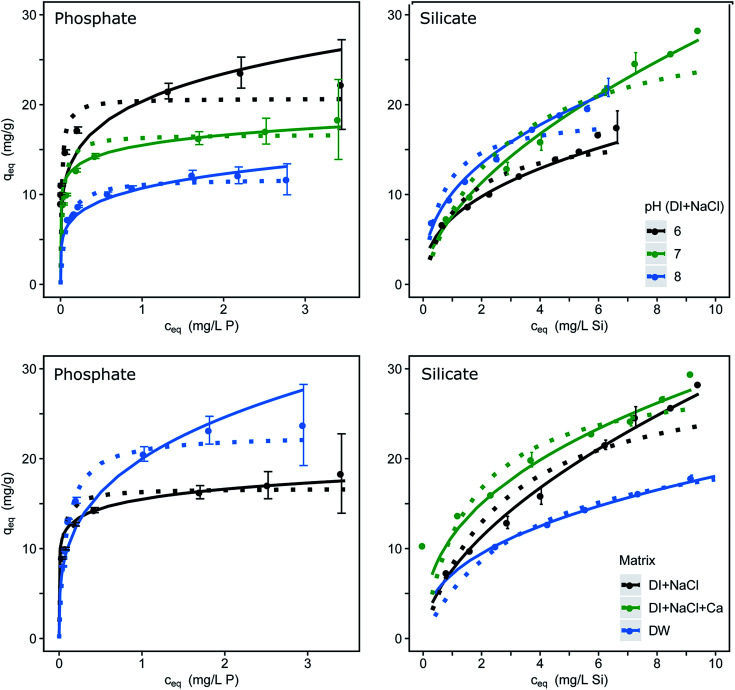
Adsorption isotherms dependent on the pH (upper row) and on the water matrix (lower row) for phosphate and for silicate; errors indicate the 95% confidence interval; Freundlich isotherms as continuous and Langmuir as dotted lines fitted, with constants for pH 7.0 and DI provided in Table 1. Redlich–Peterson isotherm fittings are found in Fig. S1.†

The single-solute adsorption isotherms for phosphate and silicate are dependent on the pH value and the water composition as shown in Fig. 1. Fitting parameters were obtained through linearization of the single-solute models and are listed in Table 1. The μGFH surface sites with a pH_PZC_ of 7.5 react as acid or base dependent on the pH.^25^ At pH values below the pH_PZC_ the μGFH surface is positively charged and therefore the adsorption of anions is electrostatically favored. In the pH range of 6.0 to 8.0 phosphate is present as HPO_4_^−^ and H_2_PO_4_^2−^ (p*K*_a_ = 7.2) and thus adsorption capacity is higher for lower pH values. As the pH increases, more surface groups of the adsorbent are uncharged and negatively charged. Phosphate becomes twofold negatively charged. Thus the adsorption capacity decreases for pH values above the pH_PZC_. Several studies revealed that phosphate forms strong inner-sphere complexes such as bidentate, binuclear or monodentate surface complexes with ferric (hydr)oxide.^6,13,37^

**Table 1:** Isotherm parameters for the removal of phosphate and silicate by μGFH (pH 7.0, DI). A complete list can be found in Table S1

	Langmuir			Freundlich			Redlich–Peterson			
Adsorbate	*K*_L_ (L mg^−1^)	*q*_m_ (mg g^−1^)	*R* ^2^	*K*_F_ (L*^n^* (g mg*^n^*^−1^)^−1^)	*n* (—)	*R* ^2^	*A* (L g^−1^)	*b* (L mg^−1^)^*β*^	*β* (—)	*R* ^2^
Phosphate	40.00	16.70	0.90	15.50	0.14	0.99	500.00	30.30	0.95	0.99
Silicate	0.60	28.00	0.95	10.50	0.42	0.99	60.00	4.70	0.65	0.99

Silicate (p*K*_a_ = 9.8) loadings increase with increasing pH, as shown in Fig. 1. Also, the percentage of polymeric (mostly dimeric) silicate rises with increasing pH. Davis *et al.*^38^ suggested a direct sorption mechanism of monomeric and dimeric silicate to iron oxide surfaces, which leaves the surface with a net positive charge up to loadings of 1 mol mol^−1^ of adsorbent. The resulting negative surface charge acts electrostatically on the remaining free surface groups by increasing the fraction of protonated groups.^13^ This explains the high loadings of 28 mg g^−1^ at initial concentrations of 10 mg L^−1^ silicate.

### 3.2. Effect of water composition

P loadings of approx. 17 mg g^−1^ were reached at an equilibrium concentration of 3 mg L^−1^ P at pH 7.0 with an ionic strength of 10 mmol L^−1^. The adsorption in drinking water shows higher capacities with approx. 23 mg g^−1^ P. Several studies have shown that calcium, which occurs in concentrations up to 100 mg L^−1^ in Berlin drinking water, enhances the adsorption of phosphate^7,10^ due to a suggested multi-layer coverage or the formation of ternary surface complexes. Furthermore, the possibility of surface precipitation of calcium phosphate was discussed.^10,39^ Contrasting effects can be seen in the adsorption of silicate. In drinking water μGFH shows much lower silicate loadings than in DI water (17 mg g^−1^ compared to 27 mg g^−1^ at *c*_e_ = 9 mg L^−1^). The addition of Ca^2+^ to DI water with NaCl led to a rise in adsorption of approx. 15%. The adsorption of calcium leads to a more positive surface charge of the adsorbent, resulting in higher adsorption capacities of silicate. Thus, the reduction in DW is linked to the presence of other competing anions. Bicarbonate was identified as main competitor for chromate adsorption onto μGFH^40^ and might also adversely impact silicate adsorption.

### 3.3. Reversibility of adsorption

**Fig. 2:**
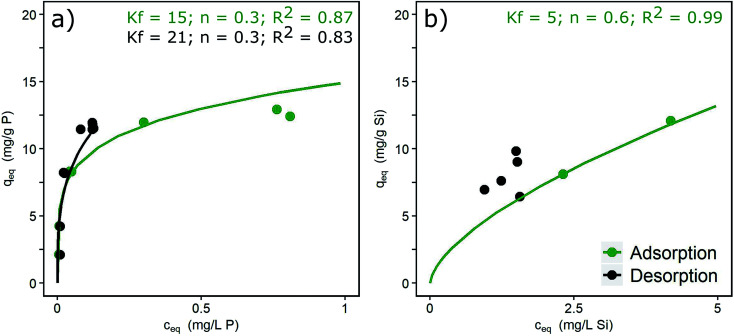
Ad- and desorption isotherms for (a) phosphate (*c*_0_ = 3 mg L^−1^) and (b) silicate (*c*_0_ = 10 mg L^−1^) (pH 7.0, DI + NaCl) and Freundlich constants.

The desorption isotherms of phosphate and silicate in DI + NaCl water are shown in Fig. 2, together with Freundlich isotherm fittings. In the case of phosphate (Fig. 2a), adsorption is partly reversible as indicated by partially overlapping ad- and desorption isotherms. An irreversible portion of adsorbed phosphate can also be observed, shown by the desorption isotherm being located above the adsorption isotherm around liquid-phase concentrations of >0.1 mg L^−1^. Similar results with 16% of irreversible bonds and slow re-diffusion out of micropores were described by Cornell and Schwertmann.^37^ Atkinson *et al.*^41^ explained the irreversibility by the formation of inert binuclear surface complexes. The adsorption isotherm of silicate is only partially shown in Fig. 2b. Independent of the loading reached during the adsorption phase, silicate desorbs down to an equilibrium concentration of approx. 2 mg L^−1^ in the desorption solution. Thus, high loadings remain in the adsorbent. High surface concentrations and contact times of silicate lead to the formation of oligomers and polymers and irreversible bonds to the hydroxide surfaces. This process is favoured by the neutral pH range.^13^ Padungthon and SenGupta^19^ regenerated a silicate loaded fixed-bed adsorber and state that desorption of silicate is more difficult due to polymerization. The mole ratios applied in the present study (Si/Fe = 0.3 to 0.6) exceeded by far the ratio above which polymerization occurs (Si/Fe > 0.1).^20^ Thus, the irreversible formation of Si–O–Si bonds is likely.

### 3.4. Multi-component adsorption modelling

**Fig. 3:**
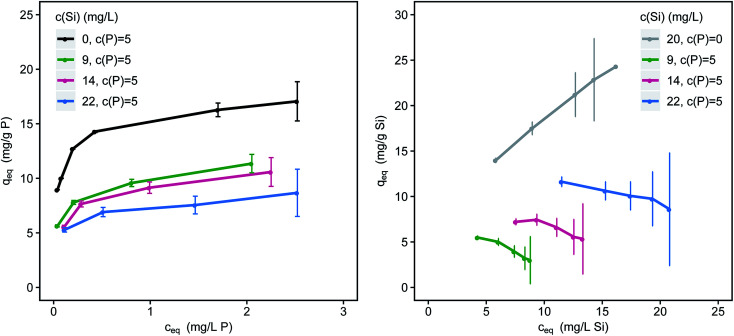
Simultaneous adsorption of phosphate (left) and silicate (right) onto μGFH with increasing concentrations of silicate (pH 7.0, DI + NaCl); error bars indicate the 95% confidence interval.

Silicate competes with phosphate for adsorption sites and reduces P-loadings significantly (Fig. 3). Phosphate loadings are reduced by 27, 33 and 47% (*c*_eq_ = 1.5 mg L^−1^) for 9, 14 and 22 mg L^−1^ Si respectively. The difference in loadings between the single-solute and the multi-component systems increases with decreasing adsorbent doses (and increasing residual P-concentrations). Sorption competition is strongly influenced by kinetics and the sorption kinetics of phosphate are considerably faster than the kinetics of silicate.^40^ The fraction of oligomeric silicates on the surface of the adsorbent steadily increases with time and loading.^18^ Especially at high loadings, silicate strongly competes with phosphate for limited adsorption sites. Silicate adsorption is also strongly affected by the presence of phosphate (Fig. 3). Through the addition of 5 mg L^−1^ P the slope of the resulting isotherm changes from positive to negative and loadings are strongly reduced.

**Fig. 4:**
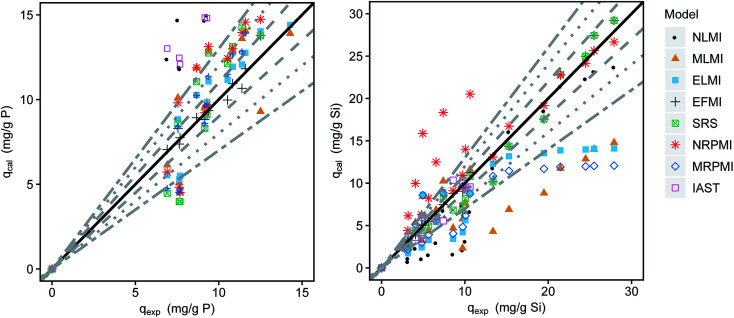
Comparison of the experimental and calculated loadings of phosphate (left) and silicate (right) in the binary mixture of phosphate and silicate (dashed lines indicate 10% deviation, dotted lines 20% and alternately dashed lines 30%).

The adsorption data of silicate and phosphate in simultaneous adsorption was fitted using the different multi-component isotherm models (eqn (4)–(13)). Comparisons between experimentally obtained and calculated loadings are shown in the parity plots (Fig. 4). The closer the data points are to the bisecting line (45° line), the better the respective model fits the experimental data. An exemplary application of the described models for the binary adsorption of phosphate and silicate with initial concentrations of 5 and 15 mg L^−1^ respectively is shown in Fig. S2† as 2D isotherms. The associated MPSD values for all tested models are given in Table 2 and the isotherms are shown in Fig. S3 and S4.†

Multi-component isotherm parameters for the simultaneous adsorption of phosphate and silicate onto μGFH

**Table:** 

Langmuir	Non-modified (NLMI)	Modified (MLMI)	Extended (ELMI)	
Fitting parameter	—	*η*_*i*_	*K*_*i*_	*q*_max_
Phosphate		12.7	62.7	14.6
Silicate		5.03	3.35	14.6
MPSD	86.0	61.0	53.3

**Table:** 

Freundlich	Extended (EFMI)			Sheindorf–Rebuhn–Sheintuch (SRS)	
Fitting parameter	*x*_*i*_	*y*_*i*_	*z*_*i*_	*a*_1,2_	*a*_2,1_
Phosphate	0.11	0.15	0.78	0.09	—
Silicate	0.78	27.3	0.41	—	713
MPSD	16.6			38.4

**Table:** 

Redlich–Peterson	Non-modified (NRPMI)	Modified (MRPMI)
Fitting parameter	—	*η*_*i*_
Phosphate		0.57
Silicate		1.11
MPSD	118	62.0

**Table:** 

Ideal adsorbed solution theory (IAST)			
Fitting parameter		*ϕ*	*q*_T_
MPSD	55.1	92–200	15.5–27.7

The non-modified Langmuir model (NMLM) creates a poor fit with a MPSD value of 80.6 as it presumes the adsorbates to adsorb independently without interaction. The introduction of the interaction term *η* results in a better fit of the MLMI (MPSD = 61.0) with a deviance of phosphate loadings below 30%, but overestimated silicate loadings. Similar fittings were achieved using the extended Langmuir model (ELMI). The MLMI uses only two fitting parameters additionally to the single-component Langmuir parameters whereas three parameters are used in the ELMI. As both models show similar fittings to the experimental data but the MLMI uses fewer fitting parameters it was chosen as the best Langmuir-type model.

**Fig. 5:**
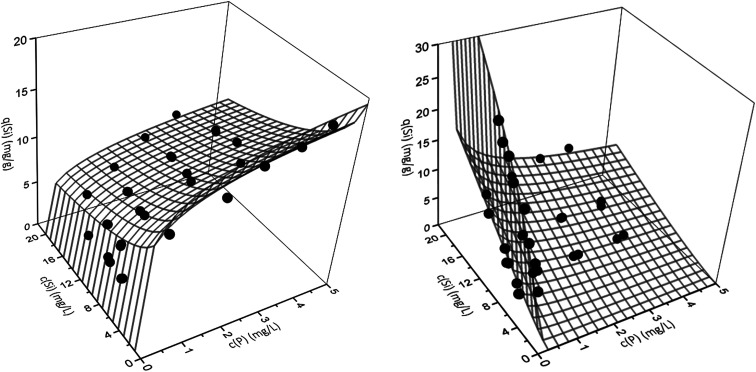
Binary adsorption isotherms for the adsorption of phosphate (left) and silicate (right) onto μGFH. Webs are predicted by the extended Freundlich model (EFMI) and symbols are experimental data.

However, the best fit with a MPSD value of 16.6 was obtained using the extended Freundlich equation assuming multilayer coverage (EFMI). This is expected as μGFH has heterogeneous surface sites and single solute isotherms were also well represented using the Freundlich model. Also, the EFMI used six fitting parameters additionally to the single-component Freundlich parameters. Thus, the interactive effects between phosphate and silicate are taken into account by the modification of the Freundlich equation. The resulting binary isotherms for phosphate and silicate are shown in Fig. 5. Phosphate loadings show a sharp increase for low phosphate or silicate concentrations, but only low additional adverse influences of silicate when its concentrations exceed 8 mg L^−1^. According to these results, even low silicate concentrations have strong negative effects on phosphate adsorption. Thus, an impact of silicate can be expected for most practical situations and water matrices. Silicate loadings are greatly reduced by the presence of phosphate, even at the lowest tested concentrations of 0.5 mg L^−1^. For P-concentrations of 1–5 mg L^−1^, the reduction of the loadings with silicate are almost constant.

The SRS model as another Freundlich derived model, but with only two fitting parameters shows a very good fit for silicate especially for higher loadings but an overestimation of up to 30% for phosphate loadings.

Both multi-component Redlich–Peterson isotherms do not reproduce the experimental values satisfactorily with errors exceeding 30% considerably. The modification with the interaction term decreases the MPSD value from 107 to 55.7.

The IAST as the only model based on thermodynamic considerations overestimates phosphate loadings by more than 30%. The poor fit (MPSD of 55.1) is explained by the heterogeneity of adsorption sites of the iron hydroxide and the interaction of the adsorbates. Both phenomena are not considered in the model calculations.

Thus, the adsorption of phosphate in competition with silicate onto μGFH can be predicted using multi-component isotherms. Freundlich derived models were found to describe the system best with MPSD values of 38.4 and 16.6 for the SRS and the EFMI. The number of fitting parameters used has to be taken into consideration.

In systems with defined matrices, resulting loadings of both adsorbents can be calculated and predicted with the described models. For drinking water (loadings shown in Fig. 1) the found correlations are not sufficient, as a multitude of water constituents affect phosphate adsorption. Thus, for complex waters, like drinking water or waste water main competitors have to be identified and modelled in order to predict the removal of certain ions.

### 3.5. Influence of contact time

**Fig. 6:**
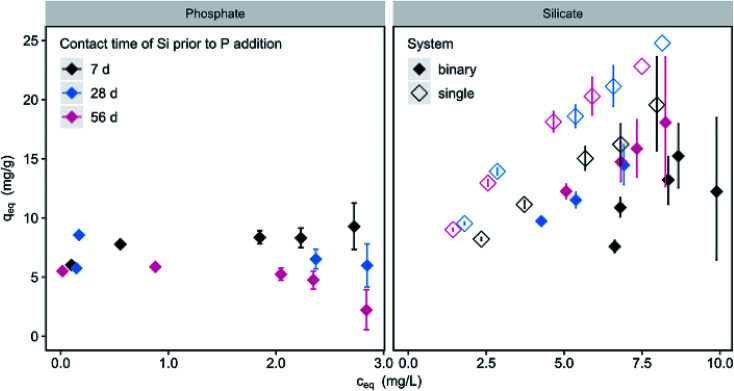
Phosphate (*c*_0_ = 3 mg L^−1^) and silicate (*c*_0_ = 10 mg L^−1^) loadings as a function of silicate contact time of 7, 28 and 56 days prior to phosphate addition (filled diamonds) in contrast to silicate as single solute (blank diamonds) (*c*(μGFH) = 150 mg L^−1^, pH 7.0, DI + NaCl).

The contact time of silicate with the adsorbent prior to the addition of phosphate influences the loadings (Fig. 6). For 150 mg L^−1^ μGFH P-loadings are reduced by 21 and 43% for 28 and 56 d compared to loadings at 7 d. No significant influence (<5%) was observed at high adsorbent doses of 600 mg L^−1^. At the same time, a significant increase (18%) of silicate loadings in the single and binary system can be observed when increasing the contact time from 7 to 28 d. No further increase was measured for 56 d. This contradicts results from Christl *et al.*^18^ who found a steady increase of silicate loadings on hematite over a time period of 210 days. Using ATR-FTIR spectroscopy, they measured a shift from monomeric to oligomeric and polymeric surface complexes over adsorption time. The competitive behaviour of chromate and silicate was studied by Zachara *et al.*^42^ Chromate adsorption was greatly reduced by the presence of silicate and the reduction increased with increasing contact time (1–7 d) of silicate with an amorphous iron hydroxide. No further changes were observed between 7 and 28 d contact time. This effect was explained by silicate polymerization in solution and on the iron surface. Smith and Edwards^43^ described the reduction of arsenate adsorption onto amorphous iron hydroxide in the presence of silicate as highly time dependent. Silicate coats the accessible surface sites within minutes of contact, resulting in an anionic surface charge. As a monomeric surface layer has a thickness of approx. 0.1 nm, physical blockage of the internal pores is unlikely at that stage.^43^ The negative surface charge however can hinder the diffusion of arsenate into the internal pores. With prolonged contact times of days and weeks, silicate forms polymers and an amorphous solid phase on the hydroxide surface with a thickness up to 100 nm, thus blocking access to internal pores and preventing arsenate adsorption.

With μGFH as adsorbent and initial silicate concentrations of 10 mg L^−1^, phosphate access to adsorption sites is not completely blocked, as can be inferred from only partially reduced phosphate loadings in Fig. 5. However, phosphate adsorption was reduced to 2.2 mg g^−1^ at low adsorbent doses and long contact times. As there is no difference in silicate adsorption between a contact time of 28 and 56 d, but phosphate adsorption is further reduced, the formation of a coating layer preventing access to internal pores and hindering adsorption is likely.

The order of addition does not lead to significantly differing loadings for phosphate or silicate at pH values of 7.0 and 8.0 at contact times of 7 d (Fig. S5†). At a pH of 6.0 however, the adsorbent surface is positively charged and thus favours anion adsorption. If phosphate (*c*_eq_ = 2 mg L^−1^) is added at a pH of 8.0 prior to silicate, resulting P-loadings are 26% higher than for later addition. Adsorption modelling using the double layer model (MINEQL5.0 with hydrous ferric hydroxide as adsorbent surface and *c*(Fe) = 76 mg L^−1^, *c*(P) = 2 mg L^−1^, *c*(Si) = 10 mg L^−1^, ionic strength = 10 mmol L^−1^) predicts Fe–HPO_4_^−^ (approx. 60%) and Fe–PO_4_^2−^ (approx. 35%) as main resulting surface species (Fig. S6†). Only at pH values above 8.5 Fe–PO_4_^2−^ predominates. Silicate adsorbs exclusively as Si(OH)_4_ up to pH 8.0. When silicate adsorbs first, it shifts the pH_PZC_ of iron hydroxides to lower values^14^ and thus impedes phosphate adsorption. Also, through the addition of silicate, previously adsorbed phosphate is replaced.

## 4. Conclusions

Competing effects of silicate strongly influence phosphate adsorption onto μGFH. Phosphate loadings on μGFH were reduced by 14, 23 and 41% in the presence of 6, 10 and 18 mg L^−1^ silicate, respectively. If the number of adsorption sites is limited, phosphate outcompetes silicate while the influence of silicate rises with increasing numbers of available adsorption sites. Thus, monitoring of silicate concentrations and adjustments of adsorbent dose in phosphate elimination processes are necessary to ensure high P-removals. In contrast to phosphate, silicate bonds are mostly irreversible due to the proposed formation of Si–O–Si bonds on the surface of ferric hydroxides. Phosphate adsorption is favoured compared to silicate at lower pH values below the pH_PZC_.

Binary isotherms could be fitted using the extended Freundlich equation assuming multi-layer coverage. Thus, resulting loadings of both adsorbates in competition can be predicted using the fitted model parameters. The models provide means to control phosphate elimination in water works applications. Real water samples contain a multicity of water constituents and additional effects are expected. However, the gained knowledge helps to understand the processes taking place in real water matrices.

Phosphate adsorption highly depends on the contact time of the iron hydroxide surface in silicate solution. An increase in contact time prior to phosphate addition from 7 to 28 and 56 d led to a decrease of 21 and 43% in P-loadings, respectively. Simultaneously, only minor silicate replacement by phosphate was observed, further underlining partially irreversible silicate adsorption. Thus, the formation of a coating layer resulting from silicate polymerization is likely to hinder access to adsorption sites reducing successful phosphate removal in applications with high silicate concentrations.

## Conflicts of interest

The authors declare no conflict of interest.

## Supplementary Material

RA-009-C9RA04865K-s001

RA-009-C9RA04865K-s002
