# Supplementary material for: Quantification and isotherm modelling of competitive phosphate and silicate adsorption onto micro-sized granular ferric hydroxide
Source: RSC Adv. 2019 Jul 30;9(41):23642–51. doi: 10.1039/c9ra04865k (PMC9069472; doi:10.1039/c9ra04865k)
Supplement: RA-009-C9RA04865K-s002 [file RA-009-C9RA04865K-s002.pdf]

Electronic Supplementary Material (ESI) for RSC Advances.
This journal is © The Royal Society of Chemistry 2019

# - Supporting Information -

Quantification and isotherm modelling of competitive phosphate and silica adsorption onto micro-sized granular ferric hydroxide

Inga Hilbrandt[1], Vito Lehmann[1], Frederik Zietzschmann[2], Aki Sebastian Ruhl[3], Martin Jekel[1]

[1]Technische Universität Berlin, Chair of Water Quality Control, Str. des 17. Juni 135, 10623 Berlin, Germany

[2]TU Delft, Water Management, Stevinweg 1, 2628 CN Delft, Netherlands

[3]Umweltbundesamt, Schichauweg 58, 12307 Berlin, Germany

Corresponding author: Inga.Hilbrandt@tu-berlin.de

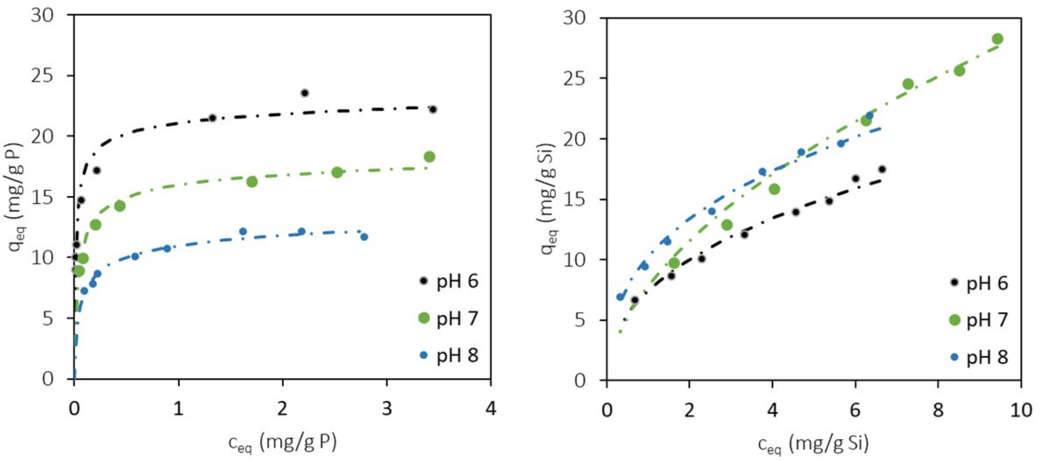

Figure S1: Redlich-Peterson isotherms for phosphate (left) and silica (right) in Di+NaCl for pH 6.0, 7.0 and 8.0.

Table S1: Isotherm parameters for the removal of phosphate and silica by μGFH

Langmuir constants

Adsorbate	Matrix	pH	K_L (L/mg)	q_m (mg/g)	R^2
Phosphate	DI+NaCl	6	69.00	20.70	0.96
	DI+NaCl	7	40.00	16.70	0.90
	DI+NaCl	8	15.00	11.80	0.92
	DW	7	12.00	22.70	0.98
Silica	DI+NaCl	6	0.84	17.50	0.91
	DI+NaCl	7	0.60	28.00	0.95
	DI+NaCl	8	1.83	18.00	0.90
	Di+NaCl+Ca	7	0.64	29.90	0.92
	DW	7	0.29	23.8	0.98

Freundlich constants

Adsorbate	Matrix	pH	K_f ((mg/g)*(L/mg)^n)	n	R^2
Phosphate	DI+NaCl	6	20.40	0.16	0.96
	DI+NaCl	7	15.50	0.14	0.99
	DI+NaCl	8	10.70	0.16	0.96
	DW	7	20.00	0.25	0.95
Silica	DI+NaCl	6	7.41	0.42	0.98
	DI+NaCl	7	10.50	0.42	0.99
	DI+NaCl	8	10.2	0.39	0.99
	Di+NaCl+Ca	7	11.4	0.40	0.97
	DW	7	7.19	0.40	0.99

Redlich-Peterson constants

Adsorbate	Matrix	pH	A (L/g)	b (L/mg)	β	R^2
Phosphate	DI+NaCl	6	1500.00	70.10	0.96	0.99
	DI+NaCl	7	500.00	30.30	0.95	0.99
	DI+NaCl	8	300.00	26.30	0.92	0.99
	DW	7	400.00	18.20	0.88	0.99
Silica	DI+NaCl	6	220.00	28.50	0.59	0.99
	DI+NaCl	7	200.00	24.50	0.45	0.98
	DI+NaCl	8	250.00	23.30	0.64	0.99
	Di+NaCl+Ca	7	200.00	15.30	0.65	0.99
	DW	7	200.00	27.10	0.61	0.99

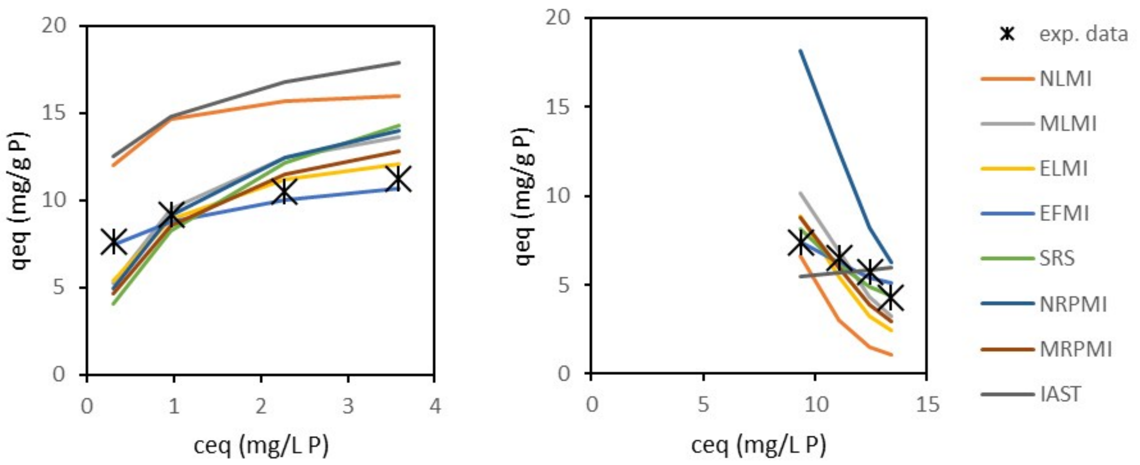

Figure S2: Application of different multi-component isotherms on binary adsorption data for phosphate (left) and silica (right) with c0(P) = 5 mg/L and c0(Si) = 15 mg/L (DI+NaCl, pH 7.0).

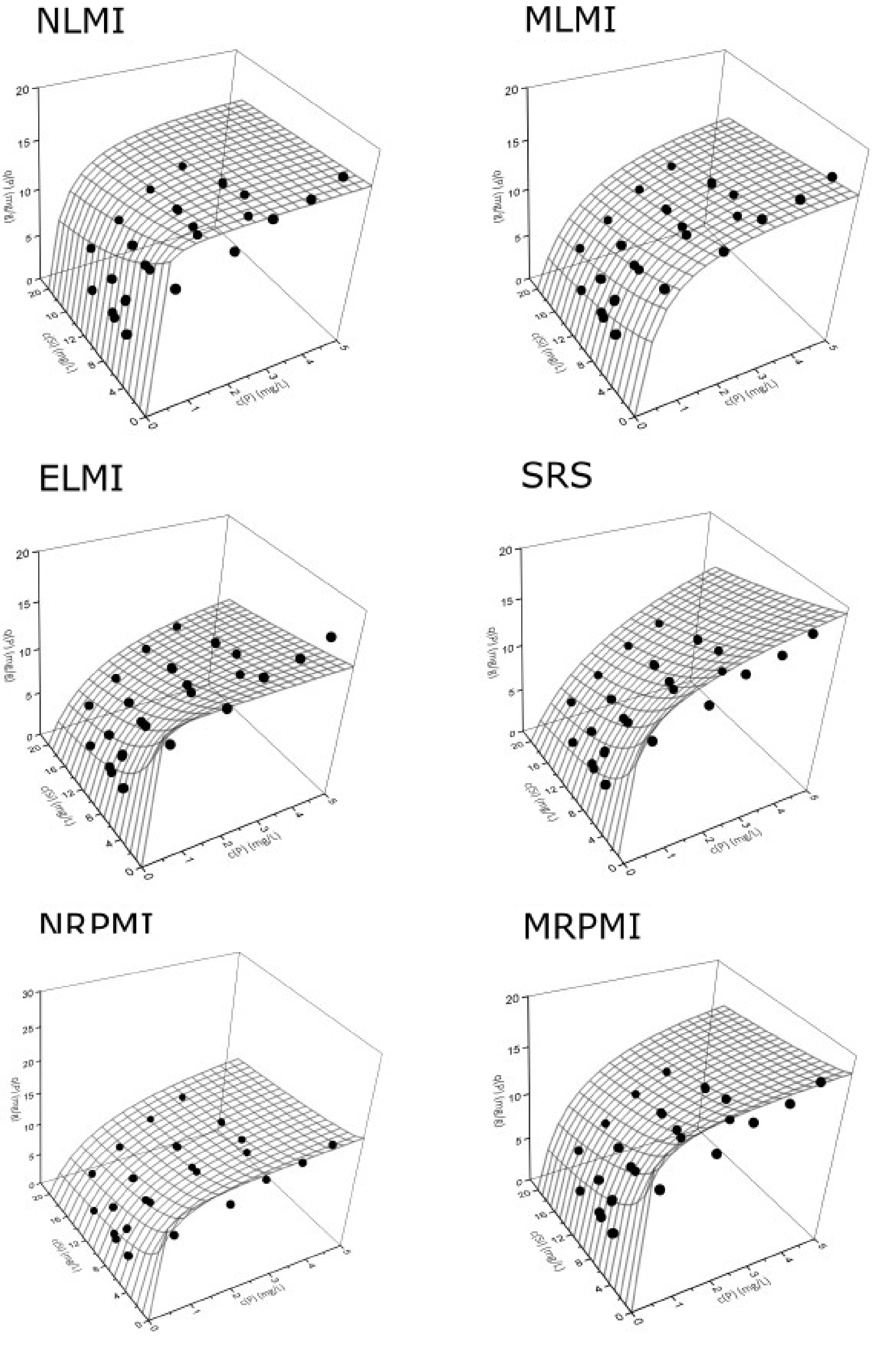

Figure 3: Multi-component isotherms for phosphate

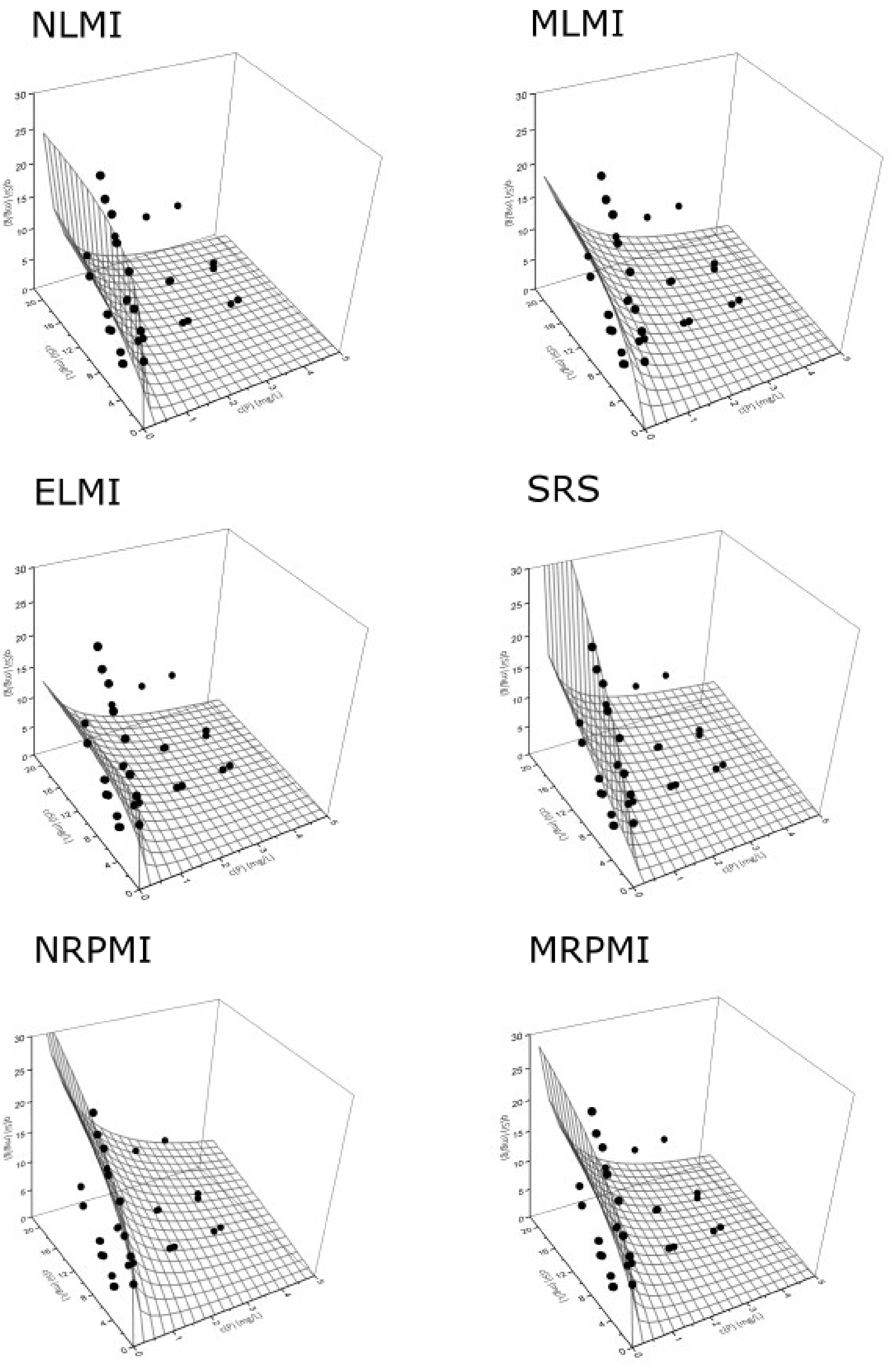

Figure 4: Multi-component isotherms for silica

Influence of order of addition

The competition between phosphate and silica is dependent on pH value and order of adsorbate addition. As the equilibrium concentration of both adsorbates differ significantly from the single component isotherm independent of pH value, competition for same adsorption sites takes place, when sites are limited. For high adsorbent doses loadings identical to mono-component adsorption were observed. As phosphate loadings decrease and silica loadings increase with increasing pH in the range 6.0 to 8.0, the influence of competition increases for phosphate with increasing pH. Phosphate loadings are reduced by 33%, 41% and 50% compared to single component adsorption for pH 6.0, 7.0 and 8.0 respectively. Also through the addition of silica, previously adsorbed phosphate is replaced. Is silica adsorbed first, it shifts the pH_{PZC} of iron hydroxides to lower values (Luxton et al. 2006) and thus complicates phosphate adsorption. P loadings were reduced by 26% (c_{eq} = 2 mg/L, pH = 6.0) in comparison to loadings with phosphate addition first. Silica adsorbs exclusively as $Si(OH)_4$ up to pH 8.0.

Another possible explanation for the decrease in adsorbed phosphate is the polymerization of silica on the iron surface which blocks adsorption sites from the contact with phosphate. For silica adsorption no influence of addition order was observed for pH 6.0. At pH 7.0 and 8.0 an increase in loadings of 15 to 28% was measured if silica was adsorbed first. Due to the induced rise in surface charge phosphate adsorption and consequently silica replacement was reduced.

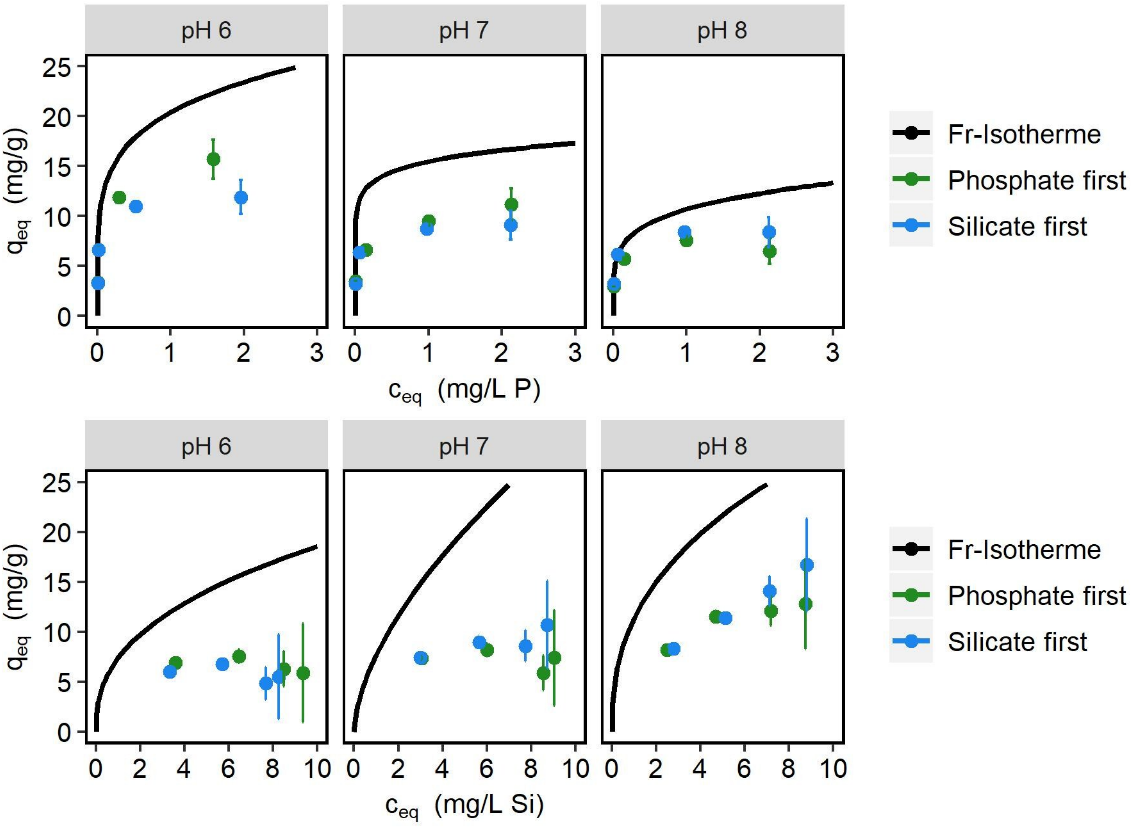

Figure S5: Adsobent loadings of phosphate and silica dependent on pH and order of addition.

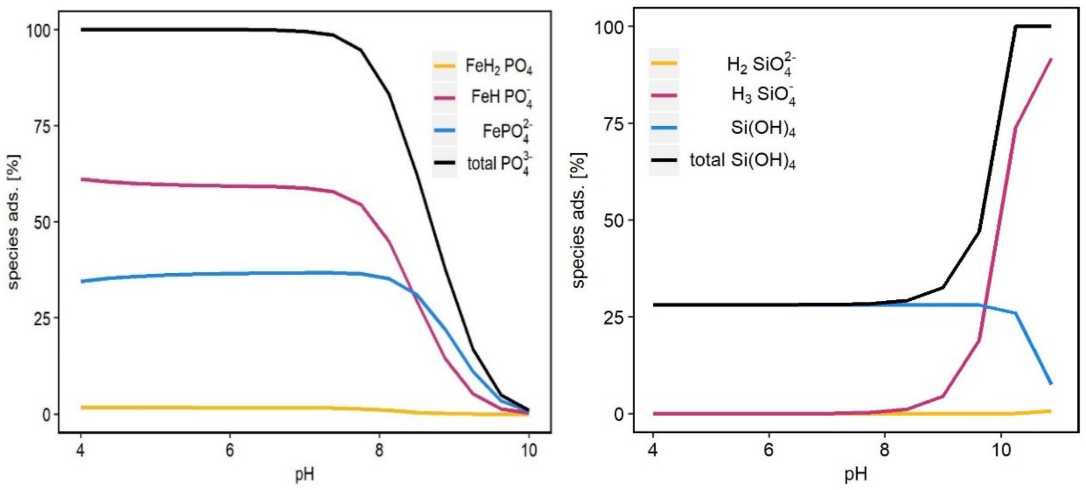

Figure S6: Species distribution of adsorbed phosphate (left) and silica (right) on ferric hydroxide dependent on pH; modelled using double layer model of Mineql5.0.
